# The long-term effect on surgery-free survival of biological compared to conventional therapy in Crohn’s disease in real world-data: a retrospective study

**DOI:** 10.1186/s12876-023-03074-x

**Published:** 2023-12-14

**Authors:** M. Valvano, A. Vinci, N. Cesaro, S. Frassino, F. Ingravalle, M. Ameli, A. Viscido, S. Necozione, G. Latella

**Affiliations:** 1 Gastroenterology Unit, Division of Gastroenterology, Hepatology, and Nutrition, Department of Life, Health and Environmental Sciences, University of L’Aquila, Piazzale Salvatore Tommasi 1, 67100 L’Aquila, Italy; 2 Hospital Health Management Area, Local Health Authority “Roma 1”, 00193 Rome, Italy; 3 University of Rome “Tor Vergata”, 00133 Rome, Italy; 4 Hospital Health Management Area, Local Health Authority “Roma 6”, 00041 Albano Laziale, Italy; 5 Area Vasta (ASUR) 5; Ascoli Piceno – San Benedetto del Tronto, San Benedetto del Tronto, Italy; 6 Epidemiology Unit, Department of Life, Health and Environmental Sciences, University of L’Aquila, L’Aquila, Italy

**Keywords:** Crohn’s disease, Biological therapy, Conventional therapy, Mesalazine, Azathioprine, Surgical resection

## Abstract

**Background:**

The introduction of biological drugs has led to great expectations and growing optimism in the possibility that this new therapeutic strategy could favourably change the natural history of Inflammatory Bowel Disease (IBD) and, in particular, that it could lead to a significant reduction in surgery in the short and long term.

This study aims to assess the impact of biological versus conventional therapy on surgery-free survival time (from the diagnosis to the first bowel resection) and on the overall risk of surgery in patients with Crohn’s disease (CD) who were never with the surgical option.

**Methods:**

This is a retrospective, double-arm study including CD patients treated with either biological or conventional therapy (mesalamine, immunomodulators, antibiotics, or steroids). All CD patients admitted at the GI Unit of the S. Salvatore Hospital (L’Aquila. Italy) and treated with biological therapy since 1998 were included in the biological arm. Data concerning the CD patients receiving a conventional therapy were retrospectively collected from our database. These patients were divided into a pre-1998 and post-1998 group. Our primary outcome was the evaluation of the surgery-free survival since CD diagnosis to the first bowel resection. Surgery-free time and event incidence rates were calculated and compared among all groups, both in the original population and in the propensity-matched population.

**Results:**

Two hundred three CD patients (49 biological, 93 conventional post-1998, 61 conventional pre-1998) were included in the study. Kaplan-Meier survivorship estimate shows that patients in the biological arm had a longer surgery-free survival compared to those in the conventional arm (*p* = 0.03). However, after propensity matching analysis, conducted on 143 patients, no significant difference was found in surgery-free survival (*p* = 0.3). A sub-group analysis showed shorter surgery-free survival in patients on conventional therapy in the pre-biologic era only (*p* = 0.02; Hazard Ratio 2.9; CI 1.01–8.54) while no significant difference was found between the biologic and conventional post-biologic groups (*p* = 0.15; Hazard Ratio 2.1; CI 0.69–6.44).

**Conclusion:**

This study shows that the introduction of biological therapy has only a slight impact on the eventual occurrence of surgery in CD patients over a long observation period. Nevertheless, biological therapy appears to delay the first intestinal resection.

## Introduction

Crohn’s Disease (CD) is an inflammatory bowel disease (IBD) characterized by a chronic disorder with periods of relapse and remission and a progressive course that leads to bowel damage and disability [1]. All segments of the gastrointestinal tract can be affected, although the terminal ileum is most frequently involved [2].

The current management of CD is focused on a treat-to-target strategy, aimed to induce a deep remission, with adjustment of appropriate medications [1, 3].

Normalization of serum and faecal markers (especially C-reactive protein and calprotectin, respectively) and achieving a condition of clinical remission are considered immediate goals that must be achieved with dose adjustment or escalation therapy. Transmural healing is a desirable target as surrogate marker of depth remission. However, currently, there is no strong evidence concerning this valuable objective in the long term [3].

Thus, in this holistic strategy, integration between conventional therapy, biological therapy, nutritional/supplementation intervention and surgery is the therapeutic tool that must be carefully selected on a case-by-case basis [1, 4–7].

The introduction of biological drugs has led to great expectations and growing optimism in the possibility that this new therapeutic strategy could favourably change the natural history of IBD and in particular, that it could lead to a significant reduction in surgery in the short and long term [8, 9].

Recently, conventional therapy has become increasingly unattractive due to its lower effectiveness in inducing and maintaining remission in the short-term period, compared to biological therapy. Moreover, meta-analyses found no evidence of the effectiveness of mesalamine (5-ASA) for the maintenance of medically induced remission in patients with CD. While 5-ASA preparations may be superior to placebo for the maintenance of surgically-induced remission in patients with CD [10, 11].

Historically, surgical resection was considered the failure of the medical treatment and, therefore, something to avoid at all costs [12]. Currently, the main indications for surgical resection are intestinal fibrostenosing obstruction, fistula, abscesses, or peritonitis [13].

Despite this, a paradigm shift concerning the surgical approach in CD is nowadays occurring. As shown in a recent randomized controlled trial (RCT), surgical resection could be a further therapeutic option in CD patients with a non-structuring, ileocecal disease and a failure to the conventional therapy, in place of infliximab therapy [14].

Data concerning the prevalence of surgical resection in CD are very heterogeneous [15–17]. Few population-based studies assessed cumulative risk of at least one intestinal resection in CD patients. In the oldest cases, the cumulative incidence of surgery among CD patients was 50% at 10 years after diagnosis; however, that result did not include data related to the biologic era [16].

An elegant population-based UK study tried to assess the cumulative probability and hazard ratios for surgery and biologic prescription from diagnosis in a Scottish population between 2000 and 2017 [18]. The 5-year cumulative risk of surgery ranged from 20.4% in the period from 2000 and 2004, to 13.0% between 2014 and 2017 (*p* < 0.001). On the other hand, the 5-year cumulative risk of biologic prescription was 5.7% in the older cohort, and 44.9% in the other cohort followed between 2014 and 2017 (p < 0.001). The authors concluded that the increased and earlier use of biologic therapy in CD patients brought a decreasing requirement for surgery over time within their cohort. However, as many authors observed, the reduction in the incidence of first intestinal surgery over time could be related to a general strategy improvement in IBD management, such as earlier diagnosis and greater patient awareness of the importance of a strict medical follow-up.

A Canadian population-based study showed that the introduction of infliximab since 1998, has not yielded anticipated reductions in the population rates of IBD-related hospitalizations or intestinal resections [17]. Moreover, a German study including 201,165 CD patients showed that the number of patients requiring surgery related to the disease remains stable over the considered period (2010–2017) [13].

It is therefore evident that the evaluation of the incidence of intestinal resection after the introduction of biological therapy is very complex and subject to several potential sources of bias, the most prominent being the shift in IBD management over the last few decades.

This study aims to assess the impact of biological versus conventional therapy on surgery-free survival time (since the diagnosis to the first bowel resection), and on overall risk of surgery in patients who were never with the surgical option.

## Methods

### Study design and population

This is a retrospective, double-arm study including CD patients treated with either biological or conventional therapy, enrolled at the IBD unit of the Gastroenterology, Hepatology and Nutrition division of the University Hospital of L’Aquila (L’Aquila, Italy). The diagnosis of CD was based on standard clinical, cross-sectional imaging techniques, endoscopic, and histological criteria [1].

All clinical investigations were conducted according to the principles laid down in the Declaration of Helsinki and reported according to the Strengthening the Reporting of Observational Studies in Epidemiology (STROBE) Statement guidelines [19]. Internal Review Board of the University of L’Aquila issued ethics approval [protocol number: IRB 58/2018.19]. All subjects gave their consent to participate in the current study and to data processing.

All CD patients treated with biological therapy since 1998 were included in the biological arm. Data concerning the CD patients receiving the conventional therapy were retrospectively collected from our database. Baseline characteristics (sex, age, date of diagnosis, disease duration) and risk factor predictors of surgery in CD (age at diagnosis, ileum-jejunal disease, active smoking status, early steroid use, disease pattern) were collected.

### Inclusion criteria

- Definite diagnosis of CD [1];
- Either:
  - ◦ Patients in biological therapy with a diagnosis made after 1998;
  - ◦ Patients on conventional therapy and with no history of biological treatments;

### Exclusion criteria

- Patients with previous intestinal resection;
- Patients with first intestinal resection in the first 6 months after diagnosis

### Outcome measures and data sources

Our primary outcome was the evaluation of the surgery-free survival (in months) since CD diagnosis to the first bowel resection, between the two following patients’ arms:

### Biological arm

We included in this group all the patients with a diagnosis of CD treated with a biological drug before their first intestinal resection. To have a more accurate representation of the real-world clinical scenario the concomitant use of immunomodulator was allowed.

### Conventional arm

We included in this group all the patients with a diagnosis of CD and treated with conventional therapy (mesalamine, immunomodulators, antibiotics, or steroid) and without a switch to the biological therapy before the outcome of interest (intestinal resection). These patients were divided into a pre-1998 and post-1998 group.

Our secondary outcome was to assess the number-needed-to-treat (NNT) among patients on biological and conventional therapy.

The following data were collected from the patients’ medical records:

- Age;
- Gender;
- Date of CD diagnosis (younger of 17 year-old was considered as early onset);
- Therapeutic regimen (biological or conventional);
- Occurrence of early steroid use (in the first year of diagnosis);
- Disease pattern (stricturing or penetrating disease behavior);
- Smoking history;
- Occurrence and date of surgery.

The main source of bias was the different indication for conventional therapy since the introduction of biological therapy: from 1998 onwards, standard therapy was reserved for the cases of mild-moderate disease. This could lead to a disease severity disproportion between the patients treated with standard therapy and those treated with biological therapy, and consequently to underestimate clinical efficacy of biological therapy. In order to mitigate the risk of deriving biased conclusions, the following strategies were employed:

- To minimize potential bias due to different risk factors associated with bad prognosis, and subsequent lower surgery-free survival, a propensity score matched analysis was performed, in addition to regular analysis.
- To minimize potential bias due to the different times of diagnosis (and thus, the different chance of undergoing conventional therapy regimen), we divided the conventional arm into two sub-groups: one including CD patients with a CD diagnosis in the pre-biologic era (≤ 1998) and one with those diagnosed in post-biologic era (≥ 1999). The 1998 cut-off date was chosen considering the market introduction of infliximab [20, 21].

### Statistical analysis

Statistical analysis was performed with Stata v. 17.0. (StataCorp. College Station, TX, USA; https://www.stata.com; 2021). Data were summarized using absolute and relative frequencies for categorical variables and median and range for numerical variables. Data were compared using the Wilcoxon and Kruskal-Wallis tests for continuous variables and Chi-Squared test for dichotomous variables.

Propensity score matching was performed using the PSMatch2 tool [22]. Propensity score for free-surgery survival was calculated using a probit regression model with the following matching variables: gender, age, early steroid use, disease pattern, smoking history, early disease onset (younger of 17 year-old) [23]. For each observation in the biologic group, up to 5 nearest neighbours in the conventional group were retained. The choice of 1:5 ratio was given since it best approximates 1:2:2 situation, given that 1:2 ratio is the recommended one for 2 group comparison [24]. Unmatched observations were excluded from the analysis. A propensity score graph was also drawn to identify and depict potential imbalance in the data.

Survivorship and event incidence rate were calculated and compared among all groups, both in original population and in propensity-matched population. Statistical significance level was set at α = 0.05 for all inferential analysis.

Kaplan-Meier function was used to estimate surgery-free survival among the three groups, measured in months since diagnosis, and respective graphs were produced [25]. The Hazard ratio (HR) among the three arms was calculated using a Cox regression model.

## Results

### Overall population

Two hundred three CD patients (49 biological, 93 conventional post-1998, 61 conventional pre-1998) were included in the study, for a cumulative period of 11,618 months. The median age was similar between the two groups (42 and 40-year-old; *p* = 0.71), however, at the end of observation periods the disease duration was longer in the biological compared to the conventional group (72 vs 36 months, respectively; *p* < 0.001).

No difference among the potential risk factors associated with a bad prognosis (risk of surgery) was found. The baseline population characteristics are summarized in Table 1. The treatment prescribed are reported in Table 2.

**Table 1:** Baseline characteristics

	Biological Arm	Conventional arm	***P***
**Population included**	49	154	***–***
**Observation time (months)**	4646	6972	0.03*
**Observed events (surgery)**	6	23	
**Incidence rate (events\year)**	0,02	0,04	
**Sex (male: n; %)**	24 (49%)	97 (63%)	0.08*
**Age at enrolment (median; range)**	42 (20–78)	40 (15–85)	0.71†
**Age at diagnosis (median; range)**	36 (9–78)	34 (11–66)	0.43^+^
**Disease duration (median; range)**	72 (2–290)	36 (12–432)	**< 0.01†**
**Early systemic steroid use**			0.73*
**‐ First year** **‐ No use in the first year**	27 (56%) 21 (44%)	91 (59%) 63 (41%)	
**Disease pattern**			0.24*
**‐ Penetrating and structuring disease** **‐ Non penetrating nor structuring disease**	21 (43%) 28 (57%)	81 (53%) 73 (47%)	
**Early onset**			0.74*
**‐ Before 17-year-old** **‐ 17–40-year-old** **‐ After 40-year-old**	3 (6%) 30 (61%) 16 (33%)	15 (10%) 92 (60%) 47 (30%)	
**Ileal or jejunum involvement**			0.39*
**‐ Yes** **‐ No**	41 (84%) 8 (16%)	136 (88%) 18 (12%)	
**Active smokers**			0.62*
**‐ Yes** **‐ No**	17 (39%) 27 (61%)	66 (43%) 88 (57%)	

*: Chi-square test; †: Wilcoxon Test; ^+^: Student’s t-test. Bold: values < 0.05

**Table 2:** Therapeutic regimen

	5-ASA	AZA	MTX	5-ASA + AZA	5-ASA + ABT	AZA + ABT	IFX^**a**^	ADA^**a**^	VEDO^**a**^	USTE^**a**^	COMBO^**a**^	SWITCH/SWAP	CCS^b^
**Biological arm**	–	–	–	–	–	–	15 (30.5%)	22 (45%)	4 (8%)	3 (6.5%)	5 (10%)	17 (35%)	49 (100%)
**Conventional arm**	8 (5%)	4 (2.5%)	4 (2.5%)	62 (40%)	38 (24.5%)	38 (24.5%)	–	–	–	–	–	–	154 (100%)

5-*ASA* Mesalamine, *AZA* Azathioprine, *MTX* Metrotexato, *ABT* Antibiotics Therapy, *IFX* Infliximab, *ADA* Adalimumab, *VEDO* Vedolizumab, *USTE* Ustekinumab, *COMBO* Combo therapy Infliximab and Azathioprine, *CCS* steroid; ^a^: first therapeutic line ^b^: at least one cycle

The treatment duration was different among the three arms (Kruskal-Wallis test *p* < 0,001).

Among patients with at least 5 years of disease duration, 8.8% (3/34) and 32.3% (21/65) of patients in biological and conventional therapy underwent surgery, respectively. The events observed during the observational period are summarized in Table 3.

**Table 3:** Study patients flow

All CD patients included: 203 patients		
**Biological arm** 49 patients **6 events** **4646 months**	**Conventional arm** 154 patients	
	61 patients diagnosed and treated before 1998	
	**13 events**	**3276 months**
	93 patients diagnosed and treated after 1998	
	**10 events**	**3696 months**
**Propensity-score matching for gender, age and risk-factor clinical variables: 143 patients**		
**Biological arm** 43 patients **6 events** **4287 months**	**Conventional arm** 100 patients	
	41 patients diagnosed and treated before 1998	
	**4 events**	**2124 months**
	59 patients diagnosed and treated after 1998	
	**6 events**	**2136 months**

*CD* Crohn’s Disease

### Propensity matched population

**Fig. 1:**
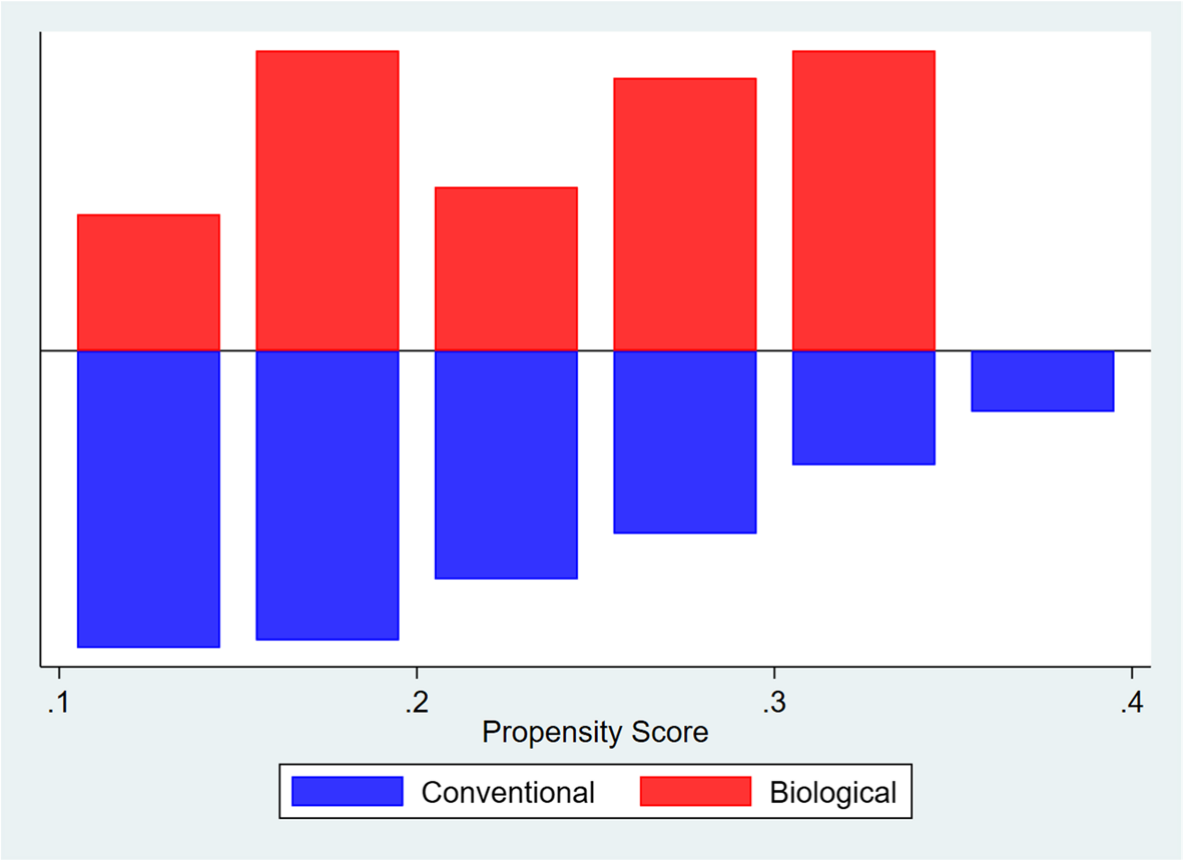
Propensity graph. Biological arm had a higher prevalence of risk factors, even after propensity adjustment

Propensity matching strategy was not particularly effective in reducing population differences between the two groups: as shown in the propensity graph (Fig. 1), patients in the biological arm were still more likely to have risk factors leading to surgery. While this is to be expected, since biological therapy is, after all, reserved to patients with more severe disease, it produced the effect of underestimating overall clinical benefit of the biological therapy in this sensitivity analysis. On the other hand, the notion that biological therapy, for severe disease, is at least as effective as conventional therapy is for mild disease, adds strength to the results we found.

After propensity score matching, 143 patients were retained (43 biological, 59 conventional post-1998, 41 conventional pre-1998), for a cumulative period of 8547 months (Table 3). The treatment duration was different among the three arms (Kruskal-Wallis test *p* < 0,001).

### Surgery incidence, surgery-free survivorship estimates and number-needed to treat

**Fig. 2:**
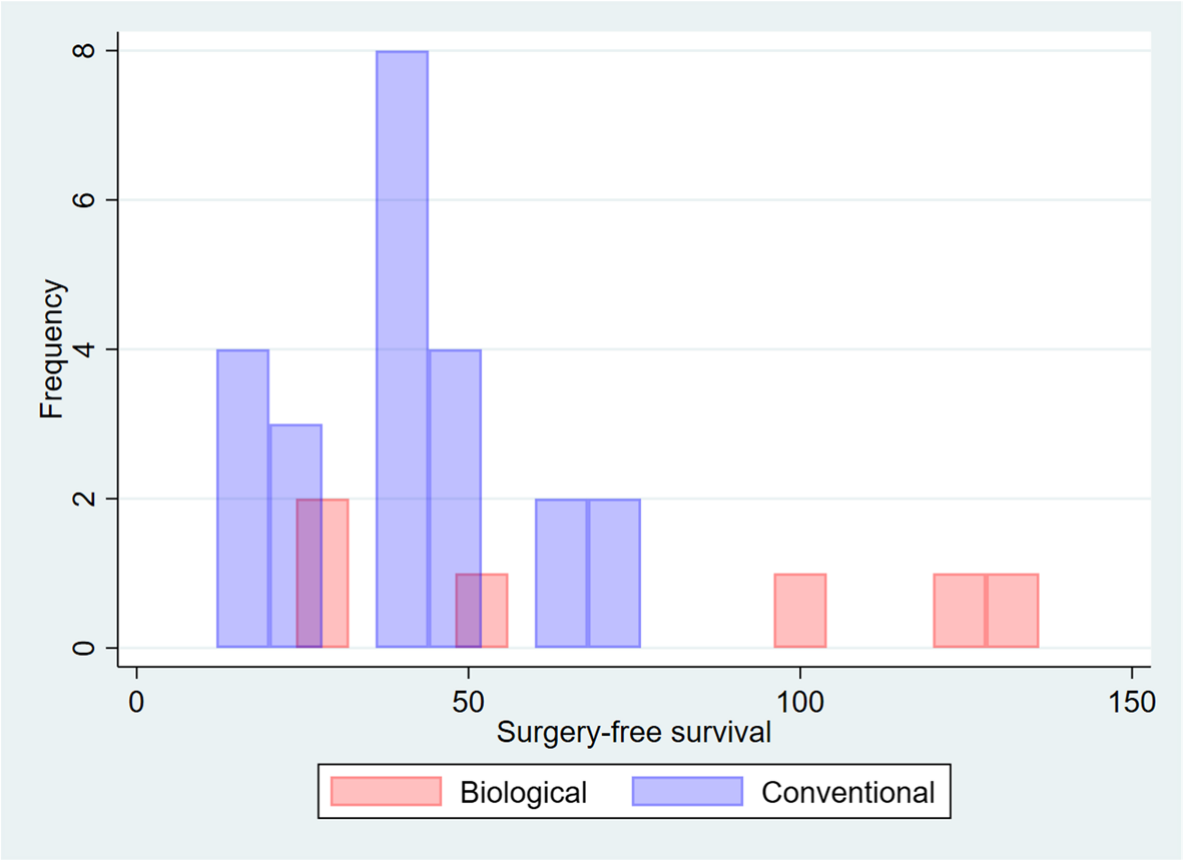
Raw distribution of surgery-free time between the two arms. Conventional arm “fails faster” to surgery than biologic arm

We observed 6 events (12%) among the 49 patients treated with biological therapy, compared to 23 (14%) among the 154 patients treated with conventional therapy. It should be noted that raw incidence alone does not account for surgery-free survival time. Even from a purely descriptive depiction such as shown in Fig. 2, conventional therapy had a higher rate of failure in the first 5 years, while biological therapy shows a flatter rate of failure, evenly spread across all the follow-up periods.

**Fig. 3:**
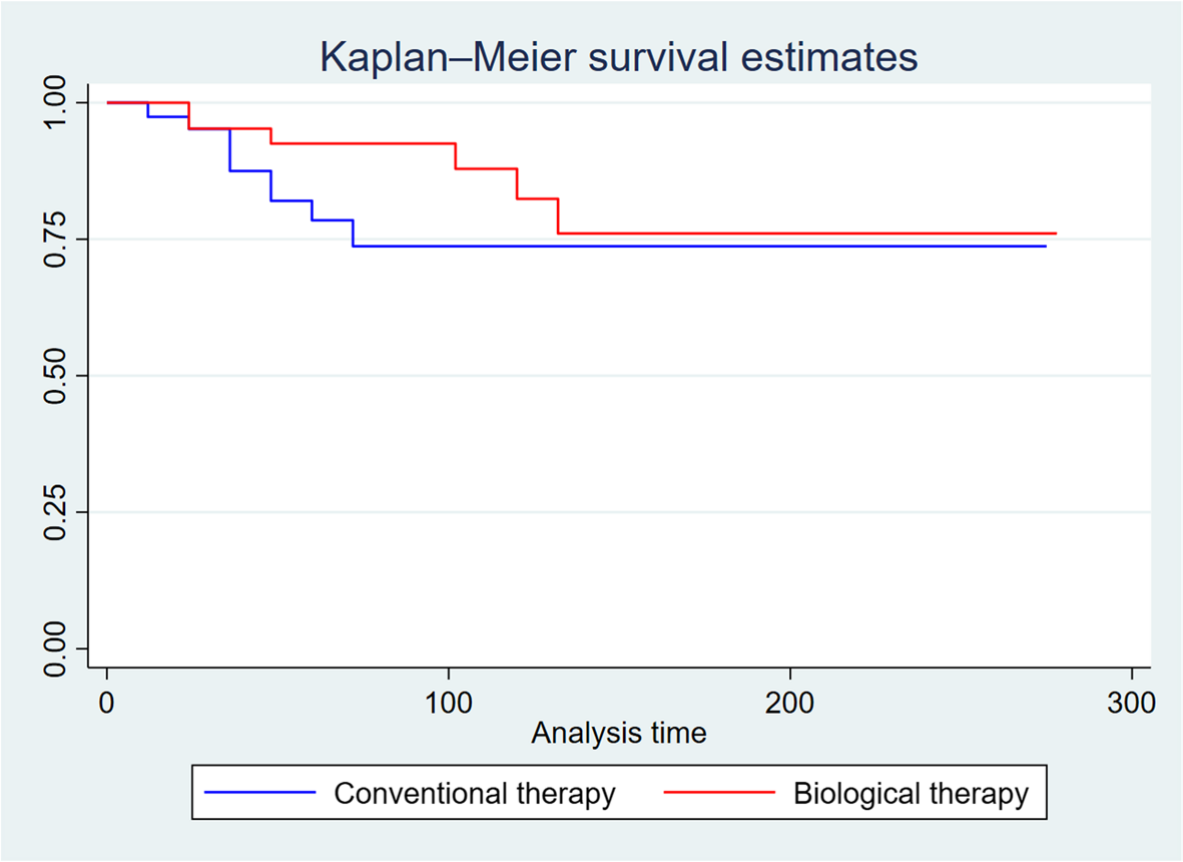
Surgery-free survival among two arms

**Fig. 4:**
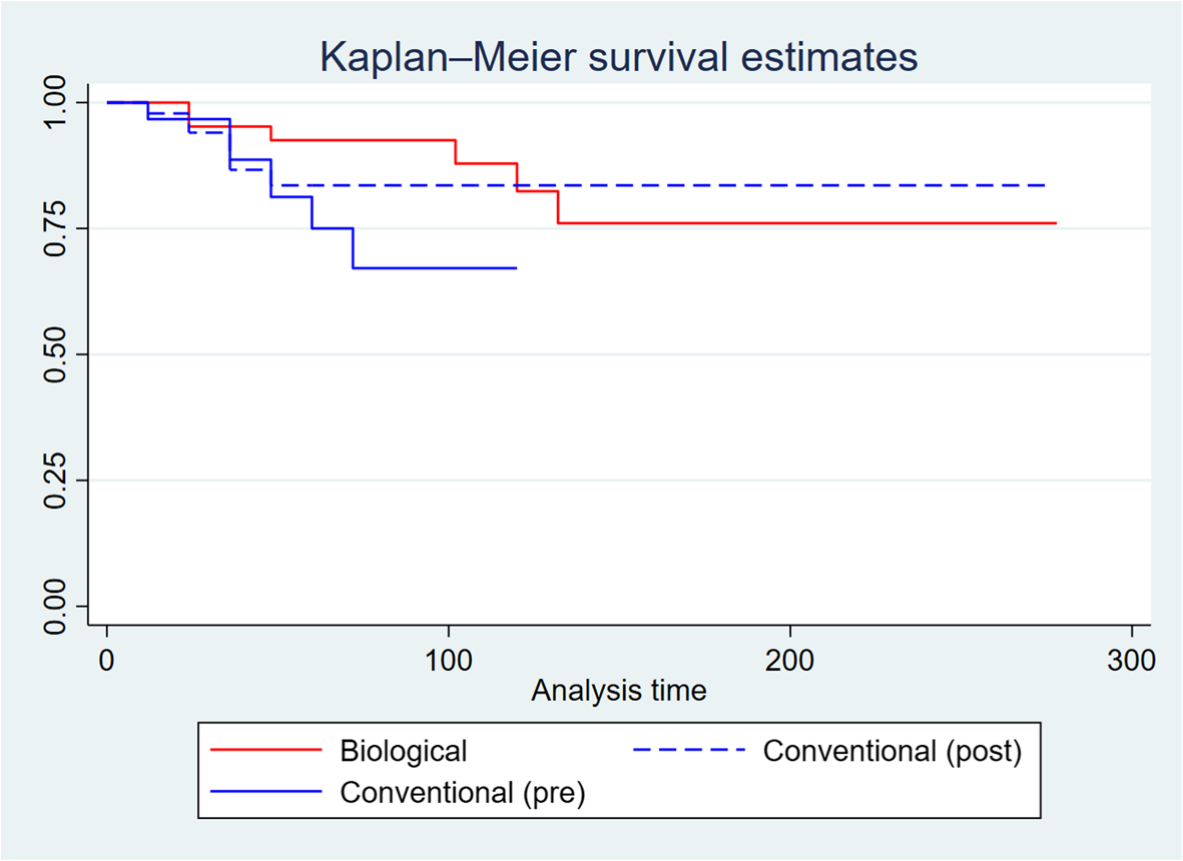
Surgery-free survival among three arms

Kaplan-Meier survivorship estimate shows that patients in the biological arm had a longer surgery-free survival compared to those in the conventional arm (*p* = 0.03; Fig. 3). However, after propensity matching analysis no significant difference was found in terms of surgery-free survival (*p* = 0.3). Figure 4 reported the surgery-free survival considering the sub-group analysis of patients on conventional therapy divided into pre- and post-biologic eras. Shorter surgery-free survival was found in patients on conventional therapy in pre-biologic era only (*p* = 0.02). Finally, no significant difference was found between the biologic and conventional post-biologic groups (*p* = 0.15; Fig. 4). At the Cox regression model among the three harms, the HR to get a surgical intervention was 2.1 (CI 0.69–6.44; *p* = 0.185) and 2.9 (1.01–8.54; *p* = 0.046) among the conventional therapy in post and pre biologic era, respectively compared to the biological therapy group.

The NNT of biological compared to conventional therapy was 37 (95% CI − 12 to ∞ to 7; *p* = 0.63).

## Discussion

Evaluation of the incidence of intestinal resection after the introduction of biological therapy is very complex and subject to several potential biases, the most prominent being the shift in IBD management over the last few decades.

In this study we have shown real-world data derived from our IBD unit, assessing the prevalence of first intestinal resection among a cohort of IBD patients treated with both biological and conventional therapies, best representing the actual clinical scenario and the disease natural history. Furthermore, to our knowledge, we reported the data with a longer observation period than usually presented in the literature. Another strength of this work is represented by the homogeneous baseline characteristic, which theoretically minimizes disproportion in the risk of surgery between the two groups. The robustness of the results is enhanced by subgroup and regression analyses.

Our study had a few important limitations. First, the inherent limits due to a non-randomized study of intervention like a retrospective design. In fact, we had limited or no information on disease severity, disease activity, or flares and we could therefore not distinguish between patients in stable remission and patients with a remittent active disease. Also, since many medical records were old and not purposely built for this study, some information such as quitting from smoke and exact symptoms onset were often unreported, or unreliable. On one hand, this made it impossible to further stratify the results by these variables. However, concerning the exact symptoms onset in an analysis timespan of more than 20 years, such as in the current work, it represents a fairly minor inaccuracy on this side. In fact, the onset of the symptoms could be approximated to the moment of diagnosis.

A potential selection bias could be identified in the different indications for conventional therapy after the marketplace introduction of biological drugs. In fact, after 1998 (the date of infliximab approval), and increasingly in recent times, more and more patients with moderate to severe disease have started biological therapy. Conversely, at least hypothetically, the conventional group with a diagnosis made after 1998 could have had a less severe disease (including only patients with a mild disease who did not need step-up therapy with the biological drugs). We found a longer surgery-free survival among patients treated with biological therapy compared to conventional therapy (Fig. 1). In propensity-matched analysis, no significant difference was found between the two arms in terms of outcome (surgery event), while biological therapy still guarantees a very long (6 years or more) surgery-free time.

Therefore, we performed a subgroup analysis to minimize potential bias due to the different times of diagnosis and thus, the different chances of undergoing the conventional therapy regimen compared to the biological treatment. We divided the conventional group into two sub-groups with a diagnosis made before or after 1998. The result of our primary outcome seems to be solid, as shown in this sub-group analysis. Only patients in conventional therapy with a diagnosis in the pre-biologic era had shorter surgery-free survival compared to patients receiving biologics (*p* = 0.02). However, no difference was found among patients in conventional therapy with a diagnosis made after 1998 and patients in biological therapy (Fig. 4). Despite the milder disease, patients in the conventional group in the biologic era had the same surgery-free survival compared to patients in biological therapy. The extremely long follow-up allows us to estimate that the prevalence of a surgical resection became similar between the two groups after about 12 years as showed in the Kaplan-Mayer analysis and as assessed by the NNT.

Interestingly, 32.3% of the patients treated with conventional therapy required the first intestinal surgery 5 years after the diagnosis similar to the data reported by Peyrin-Biroulet et Colleagues [16]. On the other hand, the 5-year cumulative risk of surgery among the biological group was 8.8%.

The effectiveness of biological therapy, that allows delaying so long the first intestinal surgery, could have major implications for the management of IBD. If the first intestinal resection is a critical issue in the IBD natural history, the postoperative recurrence (POR) and risk of multiple surgeries it is something to avoid at all cost [26]. Multiple intestinal resections could lead to a severe malabsorptive disorder such as Short Bowel Syndrome (SBS) and seriously reduced the quality of life in these patients [27, 28]. Moreover, even if few RCTs showed a lower rate of endoscopic and clinical recurrence in patients treated with anti-TNFα compared to conventional therapy we are still far from the problem solution [29–32]. In fact, POR occurs in 40% of cases within 5 years and further resections are needed in about one/third of them [2, 5, 33, 34].

This study shows that introduction of biological therapy had only a slight impact on the eventual occurrence of surgery in CD patients, over a long period of observation. Although biological therapy is able to delay the first intestinal resection, the cumulative incidence of first intestinal resection between patients who underwent biological or conventional therapy ends up being similar considering a very long period. Thus, once again a definitive treatment for the IBD treatment is far from being achieved.

## Data Availability

The datasets used and/or analysed during the current study are available from the corresponding author on reasonable request.

## Abbreviations

IBD: Inflammatory Bowel Disease
CD: Crohn’s Disease
5-ASA: 5-aminosalicylic acid
RCT: Randomized Controlled Trial
NNT: Number need to treat
POR: Post Operative Recurrence
SBS: Short Bowel Syndrome
